# Childhood maltreatment and poor functional outcomes at the transition to adulthood: a comparison of prospective informant- and retrospective self-reports of maltreatment

**DOI:** 10.1007/s00127-020-01926-5

**Published:** 2020-09-08

**Authors:** Rachel M. Latham, Emma Quilter, Louise Arseneault, Andrea Danese, Terrie E. Moffitt, Joanne B. Newbury, Helen L. Fisher

**Affiliations:** 1 Social, Genetic & Developmental Psychiatry Centre, Institute of Psychiatry, Psychology & Neuroscience, King’s College London, 16 De Crespigny Park, London, SE5 8AF UK; 2 ESRC Centre for Society and Mental Health, King’s College London, London, UK; 3 Department of Child & Adolescent Psychiatry, Institute of Psychiatry, Psychology & Neuroscience, King’s College London, London, UK; 4 National and Specialist CAMHS Trauma, Anxiety, and Depression Clinic, South London and Maudsley NHS Foundation Trust, London, UK; 5 Department of Psychology and Neuroscience, Duke University, Durham, NC USA; 6 Department of Psychiatry and Behavioral Sciences, Duke University Medical School, Durham, NC USA; 7 Center for Genomic and Computational Biology, Duke University, Durham, NC USA; 8 PROMENTA, Department of Psychology, University of Oslo, Oslo, Norway; 9 Bristol Medical School: Population and Health Sciences, University of Bristol, Bristol, UK

**Keywords:** Child abuse, Functioning, Maltreatment, Measurement, Neglect, Reporting method

## Abstract

**Purpose:**

Growing evidence suggests that prospective informant-reports and retrospective self-reports of childhood maltreatment may be differentially associated with adult psychopathology. However, it remains unknown how associations for these two maltreatment reporting types compare when considering functional outcomes. The present study compared associations between childhood maltreatment and functional outcomes at age 18 years using these two methods.

**Methods:**

We used data from the Environmental Risk (E-Risk) Longitudinal Twin Study, a nationally representative birth cohort of 2232 children born in England and Wales in 1994–1995. Maltreatment prior to age 12 years was assessed prospectively (during multiple home visits between birth and age of 12 years based on interviews with caregivers, researcher observations, and information from practitioners where child protection referrals were made) and retrospectively (at age 18 via self-report on the Childhood Trauma Questionnaire). Nine functional outcomes were measured at age 18, forming two variables capturing: (i) psychosocial and (ii) vocational disadvantage.

**Results:**

Among the 2054 participants with available data, childhood maltreatment was associated with poorer functional outcomes regardless of whether this was reported only prospectively, only retrospectively, or both. Stronger associations with psychosocial disadvantage arose in the context of retrospective recall by participants (OR = 8.25, 95% CI 4.93–13.82) than prospective reports by informants (OR = 2.03, 95% CI 1.36–3.04) of maltreatment. Conversely, associations with vocational disadvantage were comparable for both prospective informant-reports (OR = 2.19, 95% CI 1.42–3.38) and retrospective self-reports (OR = 1.93, 95% CI 1.33–2.81) of maltreatment.

**Conclusion:**

Results highlight the importance of considering the maltreatment report type used when interpreting the functional consequences of childhood maltreatment.

**Electronic supplementary material:**

The online version of this article (10.1007/s00127-020-01926-5) contains supplementary material, which is available to authorized users.

## Introduction

Exposure to child maltreatment (including neglect, physical, sexual, and emotional abuse) has consistently been associated with a wide range of adverse outcomes, including functional impairment. For example, longitudinal research suggests maltreated children have an elevated risk for being ‘Not in Employment, Education, or Training’ (NEET) [1] and lower levels of educational attainment [2]. In addition, exposure to maltreatment has been shown to increase the likelihood of criminal behaviour [3], further exposure to victimisation [4, 5], teenage parenthood [6], and is also associated with subsequent lower life satisfaction [7], loneliness [8], and poorer sleep quality [9]. Moreover, the relationship between childhood maltreatment and functional impairment appears to occur in a dose–response manner such that exposure to more forms of maltreatment confers greater risk of poor adult functioning [4, 10].

Though there is considerable evidence of the long-term adverse consequences associated with childhood maltreatment, this area of study is complicated by methodological measurement issues. Typically, studies measure participants’ exposure to child maltreatment via retrospective self-reports or prospective informant-reports. Asking adult participants to retrospectively report their experiences of maltreatment during childhood using a brief questionnaire is common, enabling researchers to assess larger samples in a timely and relatively low-cost manner. However, retrospective reporting is susceptible to a number of time-related memory biases, including memory inaccuracy due to decay [11, 12]; infantile amnesia [13] and the reconsolidation of maltreatment memories following feedback (e.g. being told something was or was not abusive) [14]. Additionally, retrospective recall of childhood maltreatment is potentially impacted by concurrent mental health. For example, individuals with depression may be more likely to remember negative than positive experiences [15, 16]. Other studies utilise prospective reports of child maltreatment; these may be from official records such as social services or healthcare records, or from caregivers. Because these reports are provided around the time of the event, they avoid the time-related problems associated with retrospective reports. The increased objectivity of this method, especially when maltreatment is independently reported by several sources, is also viewed as advantageous compared to self-reports. However, prospective informant-reports are not completely free of potential biases. For example, relying solely on official records will likely capture only the most extreme cases of maltreatment [17], thus inflating longitudinal associations with poor functioning and limiting the generalisability of findings to less severe maltreatment. The validity of prospective informant-reports may also be affected—and the association with adult outcomes underestimated—if caregivers under-report maltreatment. This may occur if they are unwilling to divulge information perhaps because they are the perpetrator, fear being referred to authorities, or they are unaware of their child’s exposure to maltreatment (e.g. if it occurs outside of the home or is secretive in nature such as sexual abuse [18]).

Studies that have collected both prospective informant-reports and retrospective self-reports of the same individual’s exposure to childhood maltreatment enable direct comparisons of these two measurement methods. Here, findings have demonstrated between-method agreement to be only low to moderate, suggesting that prospective and retrospective maltreatment reports capture two, largely non-overlapping groups of individuals [19, 20]. Moreover, there is emerging evidence that prospective informant- and retrospective self-reports may be differentially associated with adult outcomes. For example, a recent study by Newbury and colleagues [20] found that whilst both maltreatment report types predicted age-18 mental health problems, retrospective self-reports of childhood maltreatment yielded stronger associations compared to prospective informant-reports. Indeed, accounting for both report types, the relationship between childhood maltreatment and later psychopathology was found to be partly contingent on whether the maltreatment exposure was recalled in adulthood. Similar findings were also reported by Reuben and colleagues [21] in respect of childhood adversity and adult mental health. Additionally, this study examined adult physical and cognitive health outcomes and found a contrasting pattern of associations with prospective and retrospective reports according to whether these outcomes were measured objectively or subjectively. Specifically, when outcomes were objectively measured, prospective reports of childhood adversity were more strongly associated than retrospective reports but when outcomes were subjectively measured, the opposite was found—retrospective reports were more strongly associated than prospective reports [21]. The extant research therefore supports the value of both prospective informant- and retrospective self-reports of childhood maltreatment but highlights the salience of retrospective recall for adult psychopathology as well as the need to consider the type of report for adult health outcomes.

However, research to date has focused only on health and psychopathology outcomes such that it remains unknown how associations with functional outcomes compare between prospective informant- and retrospective self-reports of childhood maltreatment. Additionally, there has been little examination of the concordance (or discordance) between prospective and retrospective reports of maltreatment in association with adult outcomes. Accordingly, we had two specific aims. First, to examine whether associations between childhood maltreatment and poor functional outcomes at age 18 differed according to whether maltreatment was (i) prospectively reported by caregivers and other informants during participants’ childhood, or (ii) retrospectively reported by the participants themselves at age 18 years. Second, as individuals with concordant (i.e., both prospective informant- and retrospective self-) reports of childhood maltreatment may be more likely to have poor functional outcomes, we explored this possibility by examining associations between maltreatment and poor functioning according to whether maltreatment exposure was reported both prospectively and retrospectively, prospectively only, or retrospectively only. We hypothesised that both prospective informant-reports and retrospective self-reports of childhood maltreatment would be associated with a greater likelihood of poor functional outcomes at age 18. Furthermore, based on existing literature relating to adult mental health outcomes, we expected retrospective self-reports of childhood maltreatment to demonstrate stronger associations with functional impairment compared to prospective informant-reports.

## Methods

### Study cohort

Participants were members of the Environmental Risk (E-Risk) Longitudinal Twin Study, which tracks the development of a nationally representative birth cohort of 2232 British twin children. The sample was drawn from a larger birth register of twins born in England and Wales in 1994–1995 [22]. Full details about the sample are reported elsewhere [23] and in Supplementary Material. Briefly, the E-Risk sample was constructed in 1999–2000 when 1116 families (93% of those eligible) with same-sex 5-year-old twins participated in home-visit assessments. This sample comprised 56% monozygotic (MZ) and 44% dizygotic (DZ) twin pairs; sex was evenly distributed within zygosity (49% male). Families were recruited to represent the UK population of families with newborns in the 1990s, on the basis of residential location throughout England and Wales and mother’s age.

Follow-up home-visits were conducted when the children were aged 7, 10, 12 and 18 years (participation rates were 98%, 96%, 96%, and 93%, respectively). There were 2066 E-Risk participants who were assessed at age 18. Each participant in a twin pair was assessed by a different interviewer. There were no differences between those who did and did not take part at age 18 in terms of socioeconomic status (SES) assessed when the cohort was initially defined (*χ*^2^ = 0.86, *p *= 0.65), age-5 IQ scores (*t *= 0.98, *p *= 0.33), age-5 behavioural (*t *= 0.40, *p *= 0.69) or emotional (*t *= 0.41, *p *= 0.68) problems, or childhood poly-victimisation (*z *= 0.51, *p *= 0.61).

The Joint South London and Maudsley and the Institute of Psychiatry Research Ethics Committee approved each phase of the study. Parents gave informed consent and twins gave assent between 5 and 12 years and then informed consent at age 18.

### Measures

#### Prospective informant-reports of childhood maltreatment

Lifetime exposure to several types of maltreatment was assessed prospectively when the E-Risk participants were aged 5, 7, 10, and 12 (the age 5 assessment enquired about maltreatment since birth). Research workers visited the home in pairs and were extensively trained to detect signs of abuse or neglect. During each visit, research workers interviewed the primary caregiver (usually the mother) using a structured interview about child harm, tested the children, and observed the family environment for evidence of neglect using the Home Observation for Measurement of the Environment (HOME) [24]. Specifically, caregivers were asked several questions about whether either of their twins had been intentionally harmed (physically or sexually) by an adult or had contact with welfare agencies. If caregivers endorsed a question, follow-up questions were asked and research workers made extensive notes on what had happened and indicated whether physical and/or psychological harm had occurred. Under the UK Children Act, our responsibility was to secure intervention if maltreatment was current and ongoing. Such intervention on behalf of E-Risk families was carried out with parental cooperation in all but one case. No families left the study following intervention. An unusual feature of the E-Risk study’s assessment is that we repeatedly interviewed mothers on four occasions over the years, which allowed them to build confidence in the research team. Also, we were able to reassure mothers that if harm to the child was ongoing and had to be reported by us, reporting would be managed through a trusted familiar professional, namely the family’s registered General Practitioner. As the children grew older, some mothers who were initially reluctant to reveal abuse to us divulged details of severe abuse at a later interview.

Comprehensive dossiers have been compiled for each child with cumulative information about exposure to physical abuse by an adult; sexual abuse; physical neglect; and emotional abuse/neglect. The dossiers comprised reports from caregivers of maltreatment, recorded narratives of the caregiver interviews, recorded debriefings with research workers who had coded any indication of abuse and neglect at any of the successive home visits, and information from clinicians whenever the study team made a child protection referral. The dossiers were reviewed by two independent researchers and rated for the presence and severity (none/mild/severe) of each type of maltreatment. Inter-rater agreement between the coders exceeded 85% among the maltreatment cases, and discrepantly coded cases were resolved by consensus review. In the present study, each type of prospectively reported maltreatment was dichotomised to represent none/mild (0) versus severe (1) maltreatment. Additional details about the prospective measure of child maltreatment have been reported previously [25, 26] and are provided in the Supplementary Materials.

Given the low prevalence of some specific forms of maltreatment (e.g. sexual abuse and physical neglect) we created an ‘any maltreatment’ composite by combining all forms of prospectively reported severe maltreatment. A severe rating of physical abuse, sexual abuse, physical neglect, and/or emotional abuse/neglect equated to a severe rating of ‘any maltreatment’. Additionally, given that adversities cluster [27] and demonstrate cumulative associations with risk for poor functional outcomes in adulthood [4, 10] we created a ‘multiple maltreatment’ variable by summing and categorising all forms of severe maltreatment [range: 0 (no severe maltreatment); 1 (one form of severe maltreatment); 2 (two or more forms of severe maltreatment)]. This also takes into account the low prevalence of participants reporting three or four types of maltreatment.

#### Retrospective self-reports

**Fig. 1:**
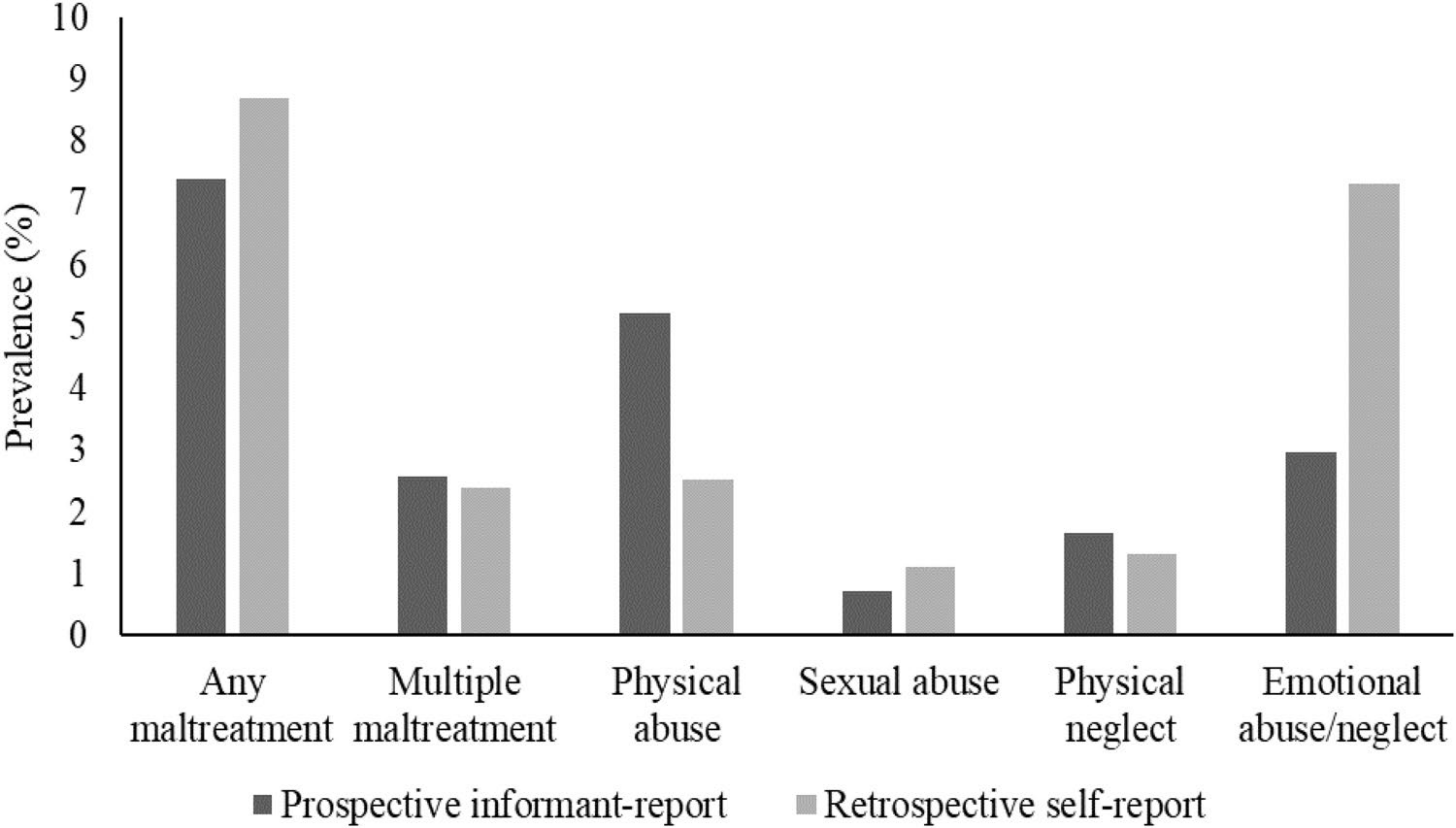
Prevalence of childhood maltreatment in the E-Risk Study according to prospective informant- and retrospective self-reports. Multiple maltreatment refers to experiencing two or more forms of maltreatment. (The E-Risk Study childhood maltreatment prevalence rates have been reported previously in Newbury et al. [20])

Maltreatment was measured retrospectively using the Childhood Trauma Questionnaire (CTQ) [28] when E-Risk participants were aged 18. The CTQ is a 25-item questionnaire used for retrospective recall of five forms of maltreatment, and has high inter-rater reliability and construct and convergent validity [29]. The CTQ is also one of the most common retrospective measures of childhood maltreatment, thus increasing the comparability of the present study with previous and future research. Participants reported on their personal experiences of physical, sexual and emotional abuse, and physical and emotional neglect for the period before they were 12 years old (i.e., before entering secondary school). Almost all (99.4%; *N* = 2054) E-Risk participants who took part in the age-18 assessment completed the CTQ. Maltreatment scores were categorised following CTQ guidelines [28] as none/minimal, low to moderate, moderate to severe, or severe to extreme. For comparability to the prospective measure of maltreatment the variable was dichotomised to represent none or minimal/low to moderate (0) versus moderate to severe/severe to extreme (1) maltreatment. To allow retrospective self-reports of maltreatment to be compared to prospective informant-reports, emotional abuse and emotional neglect were combined so that a moderate to severe/severe to extreme score for emotional abuse and/or emotional neglect represented a moderate to severe/severe to extreme score for ‘emotional abuse/neglect’. As with the prospective reports, we created ‘any maltreatment’ and ‘multiple maltreatment’ variables following the same procedure described above but using the moderate to severe/severe to extreme retrospective self-reports of maltreatment. The prevalence of childhood maltreatment within the E-Risk Study according to prospective informant-reports and retrospective self-reports is shown in Fig. 1.

#### Concordance of maltreatment reports

**Fig. 2:**
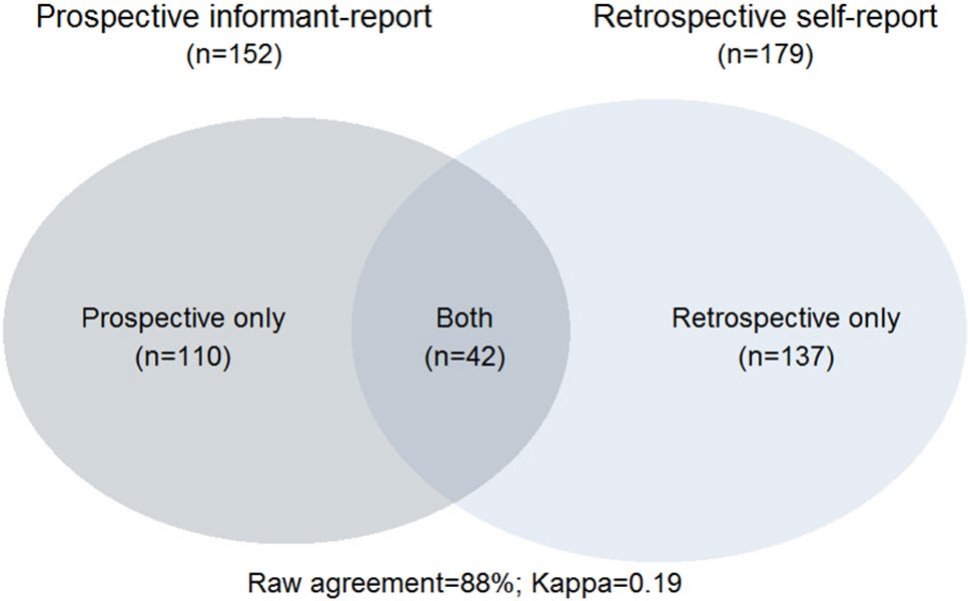
Venn diagram showing the overlap between participants exposed to any maltreatment identified by prospective informant-report and retrospective self-report measures. The light circle indicates retrospectively reported maltreatment whereas the dark circle indicates prospectively reported maltreatment. The light non-overlapping section (retrospective only) shows the number of participants who retrospectively reported a history of childhood maltreatment but were not prospectively identified as experiencing maltreatment. The dark non-overlapping section (prospective only) shows the number of participants who were prospectively reported as experiencing maltreatment in childhood but did not retrospectively self-report this maltreatment history. The overlap between the two circles shows the number of maltreated participants whose exposure was reported both prospectively and retrospectively

To take account of the corresponding maltreatment report type, we created a dummy-coded variable that captured the concordance (or discordance) between each participant’s prospective and retrospective report of any maltreatment [coded 0 (neither report maltreatment); 1 (prospective informant-report of maltreatment only); 2 (retrospective self-report of maltreatment only); 3 (both reports of maltreatment)]. Figure 2 shows the number of maltreated participants identified by both reports, prospective only, and retrospective only. Full details of the agreement between prospective informant-report and retrospective self-report measures of childhood maltreatment in the E-Risk Study have been previously reported and indicate slight to fair between-method agreement (all Kappa’s ≤ 0.31; see Newbury et al. [20]).

#### Age-18 functional outcomes

We assessed nine functional outcomes at age 18 years (see Table 1 and detailed descriptions in the Supplementary Material). Cautions and convictions were assessed through the UK Police National Computer record searches; all other outcomes were assessed at the age-18 interview. NEET status, parenthood, and cautions and convictions were naturally dichotomous; all other variables were dichotomised. For variables with no predetermined cut-off (social isolation, low life satisfaction, loneliness, and low sleep quality) we defined poor functioning as being among the 20% worst scoring participants for an outcome (as previously used in this cohort [30, 31]).

**Table 1:** Summary of age-18 functional outcome measures

Outcome	Description
Low educational achievement^a^	Did not obtain any school-leaving qualification(s) or only at low grades (GCSE grades D–G) and did not obtain any A-levels
NEET status	Participant is classified as NEET if not studying, nor working in paid employment, nor pursuing a vocational qualification or apprenticeship
Parenthood	Any past live birth or current pregnancy (girls) or having caused a pregnancy that resulted in a live birth (boys)
Cautions and convictions	Official record of any UK caution or convictions, beginning at age 10 years, the age of criminal responsibility
Adolescent poly-victimisation	Experience of two or more types of victimisation between ages 12 and 18 years. Assessed using the JVQ, adapted as a clinical interview covering 7 categories of victimisation: crime victimisation, peer/sibling victimisation, cyber-victimisation, sexual victimisation, family violence, maltreatment, and neglect
Social isolation	High score (among the top 20% of participants) on the Multidimensional Scale of Perceived Social Support. Reverse-coded to assess social isolation
Low life satisfaction	High score (among the top 20% of participants) on the Satisfaction with Life Scale, reverse-coded to assess low life satisfaction
Loneliness	High score (among the top 20% of participants) on the UCLA Loneliness Scale
Low sleep quality	High score (among the top 20% of participants) on the Sleep Quality Index

*GCSE* General Certificate of Secondary Education; *NEET* Not in Employment, Education or Training; *JVQ* Juvenile Victimization Questionnaire

^a^In the United Kingdom, students are eligible to leave school upon completion of the GCSE examination at age 16 years. Some students remain in school for an additional 2 years to complete Advanced level (A-level or equivalent) qualifications, which are required for university entrance. Participants with poor educational qualifications were those who did not obtain their A-level qualifications, scored a low grade (D–G) on their GCSE examinations or no GCSEs. See Supplementary Material for full details of measures and references

For data reduction, we performed exploratory factor analysis (principal factors with varimax rotation). Because our variables are dichotomous, this was performed on the polychoric correlation matrix. Results suggested a two-factor solution based on eigenvalues greater than 1 (Supplementary Table S1). We then ran a confirmatory factor analysis (maximum-likelihood factor method with orthogonal rotation) specifying two factors (Supplementary Table S2). The first factor—conceptualised as ‘psychosocial disadvantage’—comprised adolescent poly-victimisation, social isolation, low life satisfaction, loneliness, and low sleep quality. The second—conceptualised as ‘vocational disadvantage’—comprised low educational achievement, NEET status, parenthood, and criminal cautions and convictions. Factor analyses were repeated with the sample split randomly in half and the exploratory and confirmatory analyses conducted in separate parts of the sample. Results were comparable. Accordingly, we created two age-18 outcome variables capturing whether individuals had any: (i) psychosocial disadvantage (1 = yes; 0 = no), or (ii) vocational disadvantage (1 = yes; 0 = no). These functioning factors have been used previously in this cohort to minimise multiple testing [30].

#### Confounders

Sex of the child was reported by mothers at study baseline.

IQ was assessed at age 5 using the Vocabulary and Block Design subtests of the Wechsler Preschool and Primary Scale of Intelligence-Revised [32]. IQs were prorated (i.e., the full-scale IQ score was estimated from two subscales) following procedures described by Sattler [33] and then standardised with a mean of 100 and a standard deviation of 15.

Family socioeconomic status (SES) was defined at age 5 using a standardised composite of parental income (i.e., total household income), education (i.e., highest parent qualification), and occupation (i.e., highest parent occupation). These three SES indicators were highly correlated (*r* = 0.57–0.67) and loaded significantly onto one latent factor [34]. The population-wide distribution of this latent factor was then divided into tertiles (i.e., low-, medium-, and high-SES).

Depressive disorder over the previous 12 months was ascertained in accordance with DSM-IV criteria [35]. Private face-to-face interviews were conducted with participants at age 18 using the Diagnostic Interview Schedule [36].

### Analytic strategy

Analyses were conducted using Stata 15. First, we used logistic regression to examine the association of prospective informant-reports and retrospective self-reports of (i) any childhood maltreatment and (ii) multiple childhood maltreatment with (i) psychosocial disadvantage and (ii) vocational disadvantage at age 18 years. Second, to take account of the corresponding maltreatment report type, the variable capturing the concordance (or discordance) between each participant’s prospective and retrospective reports of any childhood maltreatment was entered as a predictor in logistic regression models predicting (i) psychosocial disadvantage and (ii) vocational disadvantage at age 18 years.

Given potential confounding associations with child maltreatment and functional outcomes, all models controlled for child IQ, sex, and family SES. Additionally, as our sample comprised twins, we accounted for the non-independence of observations using the Huber-White variance estimator [37], which provides robust standard errors adjusted for within-cluster correlated data.

A total of 2054 E-Risk Study participants completed the CTQ at the age-18 assessment (99.4% of the participants who took part at age 18). As missing data for study variables within this sample was less than 1% (0.34% for psychosocial disadvantage; 0.97% for vocational disadvantage; 0.58% for child IQ), logistic regression models analysed complete cases.

We also conducted three sensitivity analyses. Because prospective and retrospective maltreatment variables used different thresholds of severity (i.e., ‘severe’ for prospective reports versus ‘moderate to severe/severe to extreme’ for retrospective reports) we repeated the steps described above using a stricter definition of retrospective self-reported maltreatment (i.e., ‘none or minimal/low to moderate/moderate to severe’ versus ‘severe’ maltreatment). We also repeated our main analyses replacing the dichotomised psychosocial and vocational disadvantage outcome variables with count versions (0, 1, 2, 3+ poor outcomes) using ordered logistic regression models. Finally, because concurrent depressive disorder may affect retrospective recall of childhood maltreatment, we repeated our main analyses including age-18 depressive disorder as an additional covariate in models of associations between retrospective self-reports and functional outcomes.

## Results

The associations of prospective informant-reports and retrospective self-reports of childhood maltreatment with age-18 functional outcomes, adjusting for child IQ, sex, and family SES, are displayed in Table 2. The odds of having poor functional outcomes at age 18 were elevated among individuals with reports of childhood maltreatment regardless of whether maltreatment was reported prospectively or retrospectively. Retrospective self-reports of childhood maltreatment produced significantly stronger associations with psychosocial disadvantage than did prospective informant-reports (indicated by non-overlapping confidence intervals). For example, the odds associated with psychosocial disadvantage were more than four times greater for retrospective self-reports compared to prospective informant-reports of any maltreatment. In contrast, the odds associated with vocational disadvantage were similar regardless of report type. Because the maltreatment variables included several different forms of maltreatment, we checked if any specific form was driving the association. This revealed a similar pattern (Supplementary Table S3) with all forms of maltreatment showing stronger associations with psychosocial disadvantage when retrospectively self-reported but comparable associations for both report types with vocational disadvantage.

**Table 2:**
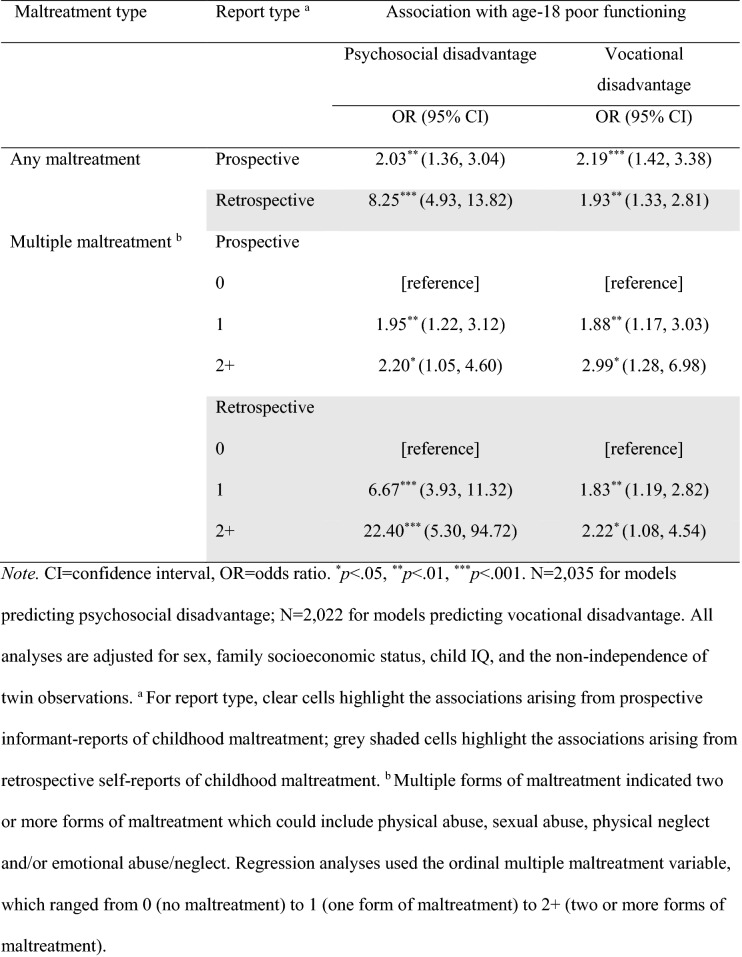
Associations of prospective informant-reports and retrospective self-reports of childhood maltreatment with age-18 functional outcomes

Table 3 shows the associations of prospective informant-report and retrospective self-reports of any childhood maltreatment with age-18 psychosocial and vocational disadvantage taking account of the concordance or discordance of the corresponding maltreatment report type. The odds of having poor functional outcomes were elevated among individuals who experienced childhood maltreatment regardless of whether they had concordant (both report types) or discordant reports (only one report type) of maltreatment. Individuals with a retrospective self-report of maltreatment but no corresponding prospective informant-report had significantly elevated odds of psychosocial disadvantage compared to those with only a prospective report of maltreatment. For example, the odds associated with psychosocial disadvantage were more than five times greater for ‘retrospective only’ compared to ‘prospective only’ reports of maltreatment. Comparing individuals with concordant reports of maltreatment with those who had discordant reports revealed a reduced likelihood of age-18 psychosocial disadvantage among those whose maltreatment was reported only prospectively (Table 3). By contrast, having only a retrospective self-report of maltreatment did not significantly alter the odds compared to having both report types.

**Table 3:**
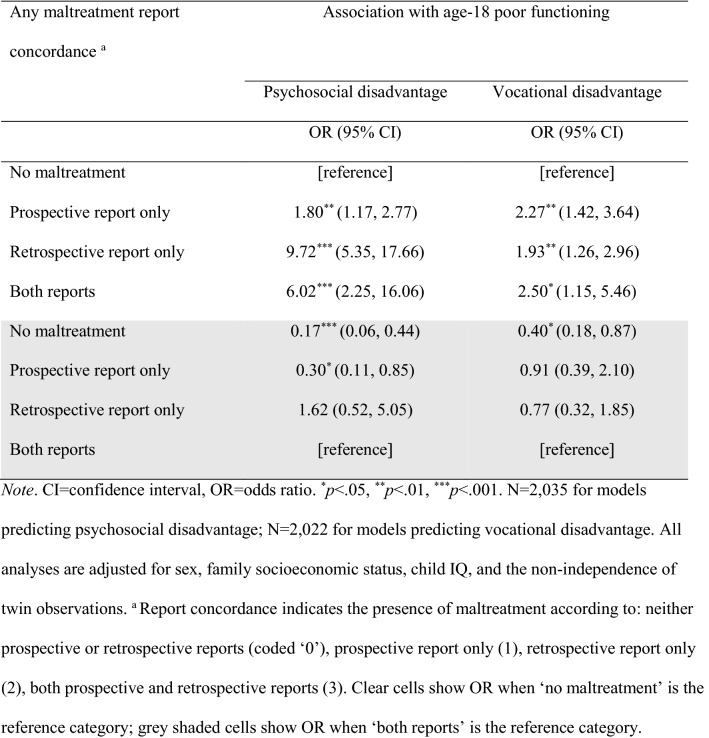
Associations of the concordance and discordance between prospective and retrospective reports of any childhood maltreatment with age-18 functional outcomes

The magnitude of the odds ratios for vocational disadvantage were similar regardless of whether there were concordant or discordant maltreatment reports (Table 3). That is, the odds associated with vocational disadvantage were around two times higher for those individuals exposed to any childhood maltreatment whether this was reported only prospectively, only retrospectively, or both.

### Sensitivity analyses

Repeating these analyses using more strictly defined retrospective self-reports of childhood maltreatment (i.e., ‘none/low/moderate’ versus ‘severe’ maltreatment) yielded a comparable pattern of results (Supplementary Table S4 and S5), suggesting that findings were not due to threshold differences between prospective and retrospective reports. Similar findings were also evident when we used a count of age-18 psychosocial and vocational disadvantage (Supplementary Tables S6 and S7). Furthermore, this same pattern of results was evident when models were adjusted for age-18 depressive disorder (Supplementary Tables S8 and S9), suggesting that findings were not due to depression impacting retrospective recall of childhood maltreatment.

## Discussion

This study extends the existing literature by comparing the associations between childhood maltreatment and age-18 functional outcomes using prospective informant-reports and retrospective self-reports of maltreatment. Our results demonstrate that childhood maltreatment is associated with increased odds of poor functioning regardless of the maltreatment report-type or the presence of a corresponding report. As expected, retrospective self-reports (compared to prospective informant-reports) yielded stronger associations with age-18 psychosocial disadvantage. However, there were no differences in their association with age-18 vocational disadvantage. We discuss these results before noting study limitations and future directions.

Consistent with a wealth of existing research [2–4, 6, 7] childhood maltreatment was associated with psychosocial and vocational disadvantage at the transition to adulthood thus supporting the notion that maltreatment constitutes a risk factor for poor functioning. This association was evident regardless of whether exposure was measured via prospective informant-reports or retrospective self-reports, and irrespective of whether there was a concordant report-type or not. This highlights the value of both report types for the prediction of functioning.

However, retrospective self-reports of maltreatment were more strongly associated with psychosocial disadvantage compared to prospective informant-reports, consistent with research pertaining to psychopathology [12, 20, 21, 38]. Notably, the odds were *most* elevated among those who retrospectively recalled childhood maltreatment but for whom there was no concordant prospective informant-report. It is possible that these individuals have disclosed maltreatment that was unknown by others during their childhood which may have led to more isolation, loneliness, possibly less access to support and, hence, a stronger association with poor psychosocial outcomes. Alternatively, it could indicate that retrospective self-reports inflate the association between childhood maltreatment and psychosocial disadvantage or, equally, that prospective informant-reports underestimate this relationship. We posit several mechanisms that may account for this potential over- or under-estimation.

First, caregivers might have withheld information about their child’s exposure to maltreatment owing to fear of referral to authorities, leading to an underestimate of the association with adolescent psychosocial disadvantage based on the prospective measure of maltreatment. This under-reporting may be especially likely if they were the perpetrator or were in a relationship with the perpetrator [18]. Considerable effort was made to reduce the likelihood of this occurring by utilising a prospective measure carefully designed to foster trust between caregivers and researchers which was also supplemented by information from other informants where relevant. On the other hand, it is possible that retrospective self-report is a better predictor of age-18 psychosocial disadvantage because participants are simply more knowledgeable about their experiences of childhood maltreatment than their caregivers or because retrospective reports were measured more proximally to the outcomes than prospective reports. However, our finding that retrospective self-reports (as compared with prospective informant-reports) did not yield stronger associations with age-18 vocational disadvantage casts some doubt on these explanations. Contrary to our hypothesis and our findings for psychosocial disadvantage, prospective and retrospective reports of maltreatment did not differ in their association with vocational disadvantage. Moreover, the magnitude of this relationship did not differ according to the presence or absence of a corresponding maltreatment report. Thus, findings derived from both prospective and retrospective reports are likely to reflect equally accurately the associations of childhood maltreatment with vocational functioning in adolescence.

In light of this, an alternative explanation for the stronger association with psychosocial disadvantage arising from retrospective self-reports may be more plausible: common method variance. Retrospective reports of maltreatment and psychosocial disadvantage were both measured concurrently via self-report which could inflate the magnitude of their association. This could occur if participants with current psychosocial difficulties define and report childhood experiences as maltreatment in an attempt to understand or explain their poor psychosocial functioning (so-called ‘effort after meaning’) [39] or if participants’ recall of childhood maltreatment is biased by depressive disorder [15]. Results of our sensitivity analyses, however, suggest that this latter mechanism of common method variance was not responsible for our findings. Conversely, the vocational disadvantage outcomes were measured using official police records and self-reports of concrete events and thus were more objective compared to the psychosocial outcome measures. Therefore, the relationship between retrospectively self-reported maltreatment and vocational disadvantage may have been less affected by common method biases.

Interestingly, our findings are only partially consistent with Reuben and colleagues’ [21] contrasting pattern of associations with prospective and retrospective reports according to whether outcomes were objectively or subjectively measured. That is, we did not find comparatively stronger associations between prospective informant-reports and vocational disadvantage. Our vocational outcomes are, however, very different from their physical and cognitive health outcomes suggesting that prospective reports will not necessarily produce stronger associations with all objective outcomes.

### Limitations

We acknowledge some limitations. First, our analyses could not separate the effects of the timing of the maltreatment report from the type of informant. As with previous comparisons of prospective informant-reports and retrospective self-reports, this unresolvable issue arises from ethical concerns that prevent studies from obtaining young children’s self-reports of their maltreatment exposure [40]. Nevertheless, we obtained the retrospective self-reports at a younger age than many previous studies [4, 41, 42] to reduce the time in which forgetting could occur. Second, the psychosocial outcomes of interest are, by their nature, most appropriately measured by self-report and therefore we could not eliminate the possibility that shared method variance impacted the association with retrospective self-reports of maltreatment. Although we control for concurrent depression, individuals who view their childhood experiences more negatively may also view their current circumstances more negatively even in the absence of a depression diagnosis. Moreover, our measure of depression captured previous year symptoms whereas identifying (and removing from the analysis) participants who were depressed at the time of the age-18 interview would have provided a more stringent sensitivity test. Third, there are other aspects of child neglect (e.g. educational and medical neglect) not captured by our prospective measure. Fourth, we used the CTQ to measure retrospective self-reports of childhood maltreatment therefore our results may not generalise to studies that utilise other retrospective report methods such as interviews. The CTQ is, however, widely used such that our findings will be relevant to a wide range of studies examining the consequences of childhood maltreatment. Fifth, our prospective and retrospective measures of childhood maltreatment differ in their operationalisation of severity with the CTQ being more focused on the frequency of maltreatment. This may limit direct comparisons of the two report types. Finally, E-Risk is a twin sample and the extent to which twin findings can be generalised to non-twins is sometimes contested (e.g. twin children may be more likely to experience maltreatment [43]). However, the prevalence of childhood maltreatment in the E-Risk cohort is comparable to recent UK population estimates [44] and the sample is representative of UK families in terms of geographic and socioeconomic distribution [45].

## Conclusion

Whilst we demonstrate the value in using both prospective and retrospective maltreatment reports for examining the functional consequences of childhood maltreatment, we highlight that using only retrospective self-reports may generate inflated associations with psychosocial disadvantage. The extent to which this observed stronger association is due to the self-reported and subjective nature of both measures, or whether individuals who recall being maltreated are genuinely at higher risk for poor psychosocial outcomes, requires further investigation. Our work contributes to the growing literature showing that prospective and retrospective measures of child maltreatment identify different people [19] and have different sets of correlates [20, 21]. Ideally, researchers should therefore use both measures of childhood maltreatment, though where this is not possible it is important to consider the type of maltreatment report being used when interpreting findings of its association with functioning.

### Statement of ethics

The Joint South London and Maudsley and the Institute of Psychiatry Research Ethics Committee approved each phase of the E-Risk study. The study has therefore been performed in accordance with the ethical standards laid down in the 1964 Declaration of Helsinki and its later amendments.

## Electronic supplementary material

Below is the link to the electronic supplementary material.

Supplementary material 1 (DOCX 61 kb)

## Data Availability

The dataset reported in the current article is not publicly available due to lack of informed consent and ethical approval for open access, but is available on request by qualified scientists. Requests require a concept paper describing the purpose of data access, ethical approval at the applicant's institution, and provision for secure data access. We offer secure access on the Duke University and King’s College London campuses. All data analysis scripts and results files are available for review.

## Code availability

Code is available on request from the corresponding author.
