# Supplementary material for: Childhood maltreatment and poor functional outcomes at the transition to adulthood: a comparison of prospective informant- and retrospective self-reports of maltreatment
Source: Soc Psychiatry Psychiatr Epidemiol. 2020 Sep 8;56(7):1161–73. doi: 10.1007/s00127-020-01926-5 (PMC8225518; doi:10.1007/s00127-020-01926-5)
Supplement: Supplementary file 1 — Supplementary material 1 (DOCX 61 kb) [file 127_2020_1926_MOESM1_ESM.docx]

**Supplementary Materials**

For Latham et al. “*Childhood maltreatment and poor functional outcomes at the transition to adulthood: A comparison of prospective informant- and retrospective self-reports of maltreatment*”

**Methods**

**Study Cohort**

Participants were members of the Environmental Risk (E-Risk) Longitudinal Twin Study, which tracks the development of a nationally representative birth cohort of 2,232 British twin children. The sample was drawn from a larger birth register of twins born in England and Wales in 1994-1995 [1]. Full details about the sample are reported elsewhere [2]. Briefly, the E-Risk sample was constructed in 1999-2000, when 1,116 families (93% of those eligible) with same-sex 5-year-old twins participated in home-visit assessments. This sample comprised 56% monozygotic (MZ) and 44% dizygotic (DZ) twin pairs; sex was evenly distributed within zygosity (49% male). Families were recruited to represent the UK population of families with newborns in the 1990s, on the basis of residential location throughout England and Wales and mother’s age. Teenaged mothers with twins were over-selected to replace high-risk families who were selectively lost to the register through non-response. Older mothers having twins via assisted reproduction were under-selected to avoid an excess of well-educated older mothers.

Follow-up home-visits were conducted when children were aged 7, 10, 12 and 18 (participation rates were 98%, 96%, 96% and 93%, respectively). Home-visits at ages 5, 7, 10, and 12 years included assessments with participants as well as their mother (or primary caregiver); the home-visit at age 18 included interviews only with the participants. Each participant in a twin pair was assessed by a different interviewer. There were 2,066 E-Risk participants who were assessed at age 18. The average age of the participants at the time of the assessment was 18.4 years (*SD*=0.36); all interviews were conducted after the 18^th^ birthday. There were no differences between those who did and did not take part at age 18 in terms of socioeconomic status (SES) assessed when the cohort was initially defined (χ^2^=0.86, *p*=0.65), age-5 IQ scores (*t*=0.98, *p*=0.33), or age-5 internalizing or externalizing behavior problems (*t*=0.40, *p*=0.69 and *t*=0.41, *p*=0.68, respectively). E-Risk families are representative of UK households across the spectrum of neighborhood-level deprivation: 25.6% of E-Risk families live in “wealthy achiever” neighborhoods compared to 25.3% of households nation-wide; 5.3% vs 11.6% live in “urban prosperity” neighborhoods; 29.6% vs 26.9% live in “comfortably off” neighborhoods; 13.4% vs 13.9% live in “moderate means” neighborhoods; and 26.1% vs 20.7% live in “hard-pressed” neighborhoods [3, 4]. Parents gave informed consent and twins gave assent between 5-12 years and then informed consent at age 18. The Joint South London and Maudsley and the Institute of Psychiatry Research Ethics Committee approved each phase of the study.

**Measures**

**Prospective informant-reports of childhood maltreatment**

Lifetime exposure to several types of maltreatment was assessed repeatedly when the children were 5, 7, 10, and 12 years of age and dossiers have been compiled for each child with cumulative information about exposure between birth and age 12 years to physical abuse by an adult; sexual abuse; physical neglect; and emotional abuse/neglect. The E-Risk team has previously reported evidence on the reliability and validity of the measures of physical and sexual abuse [5, 6], emotional abuse/neglect [7], and physical neglect [8]. All the component measures are outlined briefly below.

**Physical and sexual harm by an adult.** We assessed childhood physical and sexual harm in the E-Risk Study using an approach that resembles the process undertaken by child protection agencies. Essentially this is a two-stage process. In child protection, professionals such as teachers working with children typically raise concerns if they observe signs or symptoms or if they become aware of risk that children are victims of violence. When concerns are raised, child protection officers then review the concerns and evaluate them in the context of information previously gathered on that child or family in order to determine the likelihood that abuse has taken place. In the E-Risk Study, research workers visited the home in pairs, and were extensively trained to detect signs of abuse or neglect. Each time the two research workers visited a home, they interviewed the mother using a structured interview about child harm, tested the children, and observed the family environment using the Home Observation for Measurement of the Environment (HOME) [9]. If either research worker had any concerns, they flagged up the case for review. Immediately after each home visit, a review was performed if a family was flagged. In addition, at each wave, any family who had been flagged on a prior wave of the study was automatically reviewed again. The reviews were performed independently by at least 2 clinical psychologists or psychiatrists and were based on comprehensive dossiers compiled across multiple home visits for each study member during the course of the ongoing longitudinal study.

An unusual feature of the E-Risk study’s assessment is that we repeatedly interviewed mothers on four occasions over the years, which allowed them to build confidence in the research team. Also, we were able to reassure mothers that if harm to the child was ongoing and had to be reported by us, reporting would be managed through a trusted familiar professional, namely the family’s registered GP. As the children grew older, some mothers who were initially reluctant to reveal abuse to us, divulged details of severe abuse at a later interview.

At age 5, assessments were based on the standardized clinical protocol from the MultiSite Child Development Project [10, 11]. At ages 7, 10, and 12 this interview was modified to expand its coverage of contexts for child harm. Interviews were designed to enhance mothers’ comfort with reporting valid child maltreatment information, while also meeting researchers’ responsibilities for referral under the UK Children Act. Specifically, mothers were asked whether either of their twins had been intentionally harmed (physically or sexually) by an adult or had contact with welfare agencies. If caregivers endorsed a question, research workers made extensive notes on what had happened, and indicated whether physical and/or psychological harm had occurred. Under the UK Children Act, our responsibility was to secure intervention if maltreatment was current and ongoing. Such intervention on behalf of E-Risk families was carried out with parental cooperation in all but one case. No families left the study following intervention.

Over the years of data collection, the study developed a cumulative profile for each child, comprising the caregiver reports, recorded debriefings with research workers who had coded any indication of maltreatment at any of the successive home visits, recorded narratives of the successive caregiver interviews, and information from clinicians whenever the Study team made a child-protection referral. Each time we visited a home, the research workers flagged concerns, and if there was sufficient evidence to code definite harm then we did so. If evidence only met the level of probable harm, we kept an “ongoing concern list” and if, at a later wave, there was continued evidence of probable harm, or new evidence, the code was upgraded to definite harm. The profiles were reviewed at the end of the age-12 phase by at least two clinical psychologists or psychiatrists. Initial inter-rater agreement between the coders was 90% in cases for whom maltreatment was identified (100% for cases of sexual abuse), and discrepantly coded cases were resolved by consensus review. These were coded as: 0 = no physical harm at any age; 1 = probable physical harm at any age; and 2 = definite physical harm at any age. There were 15.0% of the children coded as probably being exposed to physical harm and 5.1% as definitely physically harmed by 12 years of age. There were 0.8% of the children coded as probably exposed to sexual abuse, and 0.7% as definitely exposed to sexual abuse by 12 years of age.

**Emotional abuse and neglect.** These forms of maltreatment were coded from research workers’ narratives of the home visits at ages 5, 7, 10, and 12. We coded quite severe examples of parental behavior observed. For example, a mother who had schizophrenia screamed and swore at the children throughout the home visit. As another example, a father who was drunk during the home visit repeatedly spoke abusively to the children in front of the research workers. We found that coders could not empirically separate emotional abuse and emotional neglect in a reliable way and thus such experiences were coded together as emotional abuse/neglect. Inter-rater agreement between the coders exceeded 85% for cases with emotional abuse and neglect, and discrepant cases were resolved by consensus review. Children with no evidence of emotional abuse/neglect were coded as 0 (88.5%), those where there was some indication of emotionally inappropriate/potentially abusive or neglectful behavior were coded as 1 (8.5%), and where there was evidence of severe emotional abuse/neglect the children were coded as 2 (3.0%).

**Physical neglect.** The cumulative observations of the physical state of the home environment documented by the interviewers during home visits to the twins at ages 5, 7, 10, and 12 were reviewed by two raters for evidence of physical neglect. This was defined as any sign that the caretaker was not providing a safe, sanitary, or healthy environment for the child. This included the child not having proper clothing or food, as well as grossly unsanitary home environments. (However, this did not include a family living in a crime-ridden neighborhood for economic reasons.) Initial interrater agreement between the coders exceeded 85%, and discrepantly coded cases were resolved by consensus review. Children with no evidence of physical neglect were coded as 0 (90.9%), those for whom there was an indication of minor physical neglect were coded as 1 (7.1%), and where there was evidence of severe physical neglect the children were coded as 2 (2.0%).

**Age-18 functional outcomes**

**Low educational achievement.** In the United Kingdom, students are eligible to leave school upon completion of the GCSE (General Certificate of Education) examination at age 16 years. Some students remain in school for an additional 2 years to complete Advanced level (A-level or equivalent) qualifications, which are required for university entrance. Participants were asked whether they had any of the following educational qualifications: 1+ GCSEs (any grades); 5+ GCSEs (grades A, B or C); A level, AS or S level, or Access Course; 2+ A levels, or 4+ AS or S levels, or any HSCs; NVQ or other vocational qualification; City and Guilds, RSA, OCR, BTEC or Edexcel; or other qualification. Based on the Qualifications and Credit Framework (QCF) responses were coded as ‘0’ no qualification; ‘1’ level 1 qualification (GCSE at grades D-G); ‘2’ level 2 qualification (GCSE at grades A*-C) or ‘3’ level 3 qualification (A-Level). In the present study low educational achievement was dichotomized to represent level 1 qualification or less (1) versus level 2/3 qualification (0). A total of 21.9% of E-Risk Study participants had low educational achievement.

**Not in Education, Employment or Training (NEET).** Participants were classified as NEET if they reported in the age-18 interview that they were neither studying, nor working in paid employment, nor pursuing a vocational qualification or apprenticeship training [12]. Participants were queried to ensure that NEET status was not simply a function of being on summer holiday, or of being a parent. This operationalization of NEET status follows that used by the UK Office of National Statistics and the International Labour Organization [13]. In the E-Risk cohort, 11.6% of participants were NEET, matching UK national NEET figures [14].

**Parenthood.** Information on parenthood was obtained in the age-18 interview by asking participants about the outcome of any previous pregnancies that they had experienced (for girls) or caused (for boys). Girls were additionally asked whether they were currently pregnant. Participants were classified as parents if a previous pregnancy had resulted in a live birth or if they were currently pregnant. 2.9% of girls and 1.1% of boys had experienced or caused at least one pregnancy that had resulted in a live birth and 0.9% of girls were pregnant at the age-18 interview. The observed rates match the UK national figures on live births for this age group [15].

**Cautions and convictions.** Official records of participants’ cautions and convictions were obtained through UK Police National Computer (PNC) record searches conducted in cooperation with the UK Ministry of Justice. E-Risk participants gave their written informed consent at age 18 years for the search of police records to be undertaken. A total of 2060 twins gave informed consent for the search at age-18 interview (99.3% of those taking part at age-18; 92.3% of the original cohort). This search was only undertaken when this consent was provided. The PNC matching algorithm, which is the same as used in law enforcement, used first, middle, and last name, date of birth, and home address. Ambiguous matches were checked by hand. Records include complete histories of cautions and convictions for participants cautioned or convicted in the UK beginning at age 10 years, the age of criminal responsibility. A criminal offence in the PNC is an offence under the Criminal Law code of the UK. Reprimands, warnings, driving offences and public order offences are not considered convictions for crimes. Cautions and convictions were recoded into a binary variable to reflect whether participants had been cautioned or convicted (1) or not (0). A total of 10.8% of E-Risk participants had been cautioned or convicted.

**Adolescent poly-victimization.** Participants reported their experiences of victimization between 12 and 18 years using the Juvenile Victimization Questionnaire (JVQ) [16, 17], adapted as a clinical interview. Full details of this measure have been reported previously [8]. In brief, our adapted JVQ comprised 45 questions covering different forms of victimization grouped into seven categories: crime victimization, peer/sibling victimization, Internet/mobile phone victimization, sexual victimization, family violence, maltreatment, and neglect. Each JVQ question was asked for the period “since you were 12”. Participants were given the option to say “yes” or “no” as to whether each type of victimization had occurred in the reporting period. If an experience was endorsed, follow-up questions were asked concerning how old the participant was when it (first) happened, whether the participant was physically injured in the event, whether the participant was upset or distressed by the event, and how long it went on for (by marking the number of years on a Life History Calendar [18]). In addition, the interviewer wrote detailed notes based on the participant’s description of the worst event. All information from the JVQ interview was compiled into victimization dossiers. Using these dossiers, each of the seven victimization categories was rated by an expert in victimology and 3 other members of the E-Risk team who were trained on using the rating criteria. Ratings were made using a 6-point scale: 0 = not exposed, then 1–5 for increasing levels of severity. Ratings of 4 or 5 were indicative of exposure to severe victimization. The number of different types of severe victimization experienced by each participant were then summed to create a score of adolescent poly-victimization (i.e. experiencing multiple types of severe victimization). We dichotomized this to reflect no poly-victimization (coded 0 = 0 or 1 type of severe victimization) versus poly-victimization (coded 1 = 2 or more types of severe victimization). A total of 16.2% of children reported adolescent poly-victimization (*n* = 334).

**Social isolation.** Social isolation was measured using the Multidimensional Scale of Perceived Social Support (MSPSS), which assesses individuals’ access to supportive relationships with family and friends [19] with 12 items consisting of statements such as “There is a special person who is around when I am in need” and “I can count on my friends when things go wrong.” Participants rated these statements as “not true” (0), “somewhat true” (1) or “very true” (2). We reversed the scoring of the items so that higher scores reflected greater isolation. We defined social isolation as being among the 20% highest scoring participants.

**Low life satisfaction.** Participants’ life satisfaction was assessed using the Satisfaction with Life Scale [20] with 5 items including “The conditions of my life are excellent” and “I am satisfied with my life”. The response format was a 5-point scale ranging from “strongly disagree” (1) to “strongly agree” (5). We reversed the scoring of the items so that higher scores reflected lower life satisfaction. We defined low life satisfaction as being among the 20% highest scoring participants.

**Loneliness.** Participants’ feelings of loneliness were assessed using four items from the UCLA loneliness scale (Version 3 [21])*:* “How often do you feel that you lack companionship?”, “How often do you feel left out?”, “How often do you feel isolated from others?” and “How often do you feel alone?” The full UCLA Scale consists of 20 items; however, a previous study has shown that a short form of the scale has adequate validity for inclusion in large-scale studies [22]. The items were rated “hardly ever” (0), “some of the time” (1) or “often” (2). The items were administered as part of a self-completed computer-based questionnaire. We summed the items to produce a total loneliness score and defined loneliness as being among the 20% highest scoring participants.

**Low sleep quality.** Sleep quality was measured using the Sleep Quality Index (PSQI [23]). The PSQI consists of 18 self-report items relating to individuals’ sleep patterns and different forms of sleep impairment in the past month. Questions tap a range of aspects of sleep quality and can be used to derive seven component scores (subjective sleep quality, sleep latency, sleep duration, habitual sleep efficiency, sleep disturbances, use of sleep medication and daytime dysfunction) each scored from 0 to 3. These were summed to produce a global score ranging from 0 to 21 with higher scores reflecting lower sleep quality. We defined low sleep quality as being among the 20% highest scoring participants.

**Table S1**

*Exploratory Factor Analysis of Functional Outcome Variables: Eigenvalues and Variance Explained Before Rotation*

| **Factor** | **Eigenvalue** | **Proportion of variance explained** | **Cumulative variance explained** |
| --- | --- | --- | --- |
| 1 | 2.93 | 0.76 | 0.76 |
| 2 | 1.18 | 0.31 | 1.07 |
| 3 | 0.25 | 0.07 | 1.14 |
| 4 | 0.09 | 0.02 | 1.16 |
| 5 | 0.03 | 0.01 | 1.17 |
| 6 | -0.06 | -0.02 | 1.15 |
| 7 | -0.15 | -0.04 | 1.12 |
| 8 | -0.19 | -0.05 | 1.07 |
| 9 | -0.25 | -0.07 | 1.00 |

*Note*. Two factors were extracted based on eigenvalues >1. The cumulative variance explained exceeds 1 (before returning to = 1) due to Factors 6, 7 and 8 having negative eigenvalues.

**Table S2**

*Confirmatory Factor Analysis of Functional Outcome Variables: Factor Loadings and Variance Explained After Rotation*

| **Functional Outcome** | **Factor 1** | **Factor 2** |
| --- | --- | --- |
| Low educational achievement | 0.56 |  |
| NEET | 0.81 |  |
| Parenthood | 0.77 |  |
| Criminal cautions & convictions | 0.55 |  |
| Adolescent poly-victimization | 0.35 | 0.47 |
| Social isolation |  | 0.63 |
| Low life satisfaction |  | 0.83 |
| Loneliness |  | 0.69 |
| Low sleep quality |  | 0.41 |
|  |  |  |
| Variance explained | 0.50 | 0.50 |

*Note*: Loadings <0.30 are not shown. NEET=Not in Education, Employment or Training. Factor 1 conceptualized as ‘vocational disadvantage’; Factor 2 conceptualized as ‘psychosocial disadvantage’. Adolescent poly-victimization cross-loaded onto both factors but was included only in Factor 2 where its loading was highest.

**Results**

**Table S3**

*Associations of Prospective Informant-Reports and Retrospective Self-Reports of Specific Forms of Childhood Maltreatment with Age-18 Functional Outcomes.*

| **Specific form of maltreatment** | **Report type ^a^** | **Association with age-18 poor functioning** | |
| --- | --- | --- | --- |
|  |  | **Psychosocial disadvantage** | **Vocational disadvantage** |
|  |  | OR (95% CI) | OR (95% CI) |
| Physical abuse | Prospective | 1.97^**^ (1.20, 3.22) | 1.96^*^ (1.18, 3.26) |
|  | Retrospective | 5.48^***^ (2.23, 13.46) | 2.07^*^ (1.03, 4.16) |
| Sexual abuse | Prospective | 2.79^†^ (0.88, 8.90) | 1.04^†^ (0.33, 3.25) |
|  | Retrospective | 19.27^**^ (2.73, 136.25) | 1.77 (0.68, 4.59) |
| Physical neglect | Prospective | 2.38^†^ (0.92, 6.12) | 3.19^*^ (1.18, 8.57) |
|  | Retrospective | 6.35^**^ (1.85, 21.74) | 1.63 (0.65, 4.08) |
| Emotional abuse/neglect | Prospective | 2.33^**^ (1.23, 4.40) | 3.29^**^ (1.50, 7.23) |
|  | Retrospective | 13.06^***^ (6.96, 24.48) | 2.22^***^ (1.47, 3.35) |

*Note*. CI=confidence interval, OR=odds ratio. N=2,035 for models predicting psychosocial disadvantage; N=2,022 for models predicting vocational disadvantage. All analyses are adjusted for sex, family socioeconomic status, child IQ, and the non-independence of twin observations. ^†^*p*<.10, ^*^*p*<.05, ^**^*p*<.01, ^***^*p*<.001. ^a^ For report type, clear cells highlight the associations arising from prospective informant-reports of childhood maltreatment; grey shaded cells highlight the associations arising from retrospective self-reports of childhood maltreatment.

**Sensitivity Analyses**

***Are findings consistent when retrospective maltreatment is defined more strictly?***

Table S4 presents results using a more strictly defined retrospective maltreatment variable (i.e. ‘none or minimal/low to moderate/moderate to severe’ versus ‘severe to extreme’ maltreatment). Elevated odds for psychosocial and vocational disadvantage were evident for individuals exposed to maltreatment regardless of report type. Furthermore, associations with psychosocial disadvantage were once again stronger for retrospective reports compared to prospective reports, though these effect size differences were less marked. The associations between childhood maltreatment and vocational disadvantage remained comparable for prospective and retrospective reports.

When retrospective reports of maltreatment were defined more strictly, a smaller number of individuals were identified as having both prospective and retrospective reports (*n* = 29). This represents 19% of the 152 individuals with prospective reports, and 32% of the 90 individuals with retrospective reports. Table S5 shows the associations between strictly defined childhood maltreatment and age-18 functional outcomes when the corresponding report type was also considered. Once again, the likelihood of poor functioning at the transition to adulthood was elevated among maltreated individuals regardless of whether this maltreatment was reported only prospectively, only retrospectively, or both. The magnitude of these associations was similar for vocational disadvantage but differed for psychosocial disadvantage. Specifically, individuals whose maltreatment was reported only retrospectively had significantly higher odds of poor psychosocial outcomes compared to those who had only prospective reports of maltreatment. In addition, there was a trend towards those individuals with only prospective reports to have lower odds of psychosocial disadvantage relative to those whose who also had a corresponding retrospective report.

**Table S4**

*Associations of Prospective Informant-Reports and Retrospective Self-Reports of Childhood Maltreatment (Using Strictly Defined Maltreatment Variables) with Age-18 Functional Outcomes.*

| **Maltreatment type** | **Report type ^a^** | **Association with age-18 poor functioning** | |
| --- | --- | --- | --- |
|  |  | **Psychosocial disadvantage** | **Vocational disadvantage** |
|  |  | OR (95% CI) | OR (95% CI) |
| Any maltreatment | Prospective | 2.03^**^ (1.36, 3.04) | 2.19^***^ (1.42, 3.38) |
|  | Retrospective | 8.37^***^ (3.86, 18.15) | 1.94^**^ (1.19, 3.18) |
| Multiple maltreatment ^b^ | Prospective |  |  |
|  | 0 | [reference] | [reference] |
|  | 1 | 1.95^**^ (1.22, 3.12) | 1.88^**^ (1.17, 3.03) |
|  | 2+ | 2.20^*^ (1.05, 4.60) | 2.99^*^ (1.28, 6.98) |
|  | Retrospective |  |  |
|  | 0 | [reference] | [reference] |
|  | 1 | 6.16^***^ (2.85, 13.32) | 1.40 (0.78, 2.51) |
|  | 2+ | 25.93^**^ (3.43, 195.99) | 3.63^**^ (1.41, 9.36) |

*Note.* CI=confidence interval, OR=odds ratio. N=2,035 for models predicting psychosocial disadvantage; N=2,022 for models predicting vocational disadvantage. All analyses are adjusted for sex, family socioeconomic status, child IQ, and the non-independence of twin observations. ^*^*p*<.05, ^**^*p*<.01, ^***^*p*<.001. ^a^ For report type, clear cells highlight the associations arising from prospective informant-reports of childhood maltreatment; grey shaded cells highlight the associations arising from retrospective self-reports of childhood maltreatment. ^b^ Multiple forms of maltreatment indicated two or more forms of maltreatment which could include physical abuse, sexual abuse, physical neglect and/or emotional abuse/neglect). Regression analyses used the ordinal multiple maltreatment variable, which ranged from 0 (no maltreatment) to 1 (one form of maltreatment) to 2+ (two or more forms of maltreatment).

**Table S5**

*Associations of the Concordance Between Prospective and Retrospective Reports of any Childhood Maltreatment (Using Strictly Defined Maltreatment Variables) with Age-18 Functional Outcomes.*

| **Any maltreatment report concordance ^a^** | **Association with age-18 poor functioning** | |
| --- | --- | --- |
|  | **Psychosocial disadvantage** | **Vocational disadvantage** |
|  | OR (95% CI) | OR (95% CI) |
| No maltreatment | [reference] | [reference] |
| Prospective report only | 1.83^**^ (1.20, 2.81) | 2.25^**^ (1.41, 3.58) |
| Retrospective report only | 11.31^***^ (4.41, 29.00) | 1.97^*^ (1.09, 3.58) |
| Both reports | 5.61^**^ (1.59, 19.88) | 2.31^†^ (0.95, 5.57) |
| No maltreatment | 0.18^**^ (0.05, 0.63) | 0.43^†^ (0.18, 1.05) |
| Prospective report only | 0.33^†^ (0.09, 1.23) | 0.97 (0.38, 2.48) |
| Retrospective report only | 2.01 (0.42, 9.69) | 0.86 (0.30, 2.47) |
| Both reports | [reference] | [reference] |

*Note*. CI=confidence interval, OR=odds ratio. N=2,035 for models predicting psychosocial disadvantage; N=2,022 for models predicting vocational disadvantage. All analyses are adjusted for sex, family socioeconomic status, child IQ, and the non-independence of twin observations. ^†^*p* <.10, ^*^*p*<.05, ^**^*p*<.01, ^***^*p*<.001. ^a^ Report concordance indicates the presence of maltreatment according to: neither prospective or retrospective reports (coded ‘0’), prospective report only (1), retrospective report only (2), both prospective and retrospective reports (3). Clear cells show ORs with ‘no maltreatment’ as the reference category; grey shaded cells show ORs with ‘both reports’ as the reference category.

***Are findings consistent when a count of age-18 psychosocial disadvantage and vocational disadvantage is used?***

Using ordered logistic regression models, we repeated our main analyses using count versions of psychosocial and vocational disadvantage (0, 1, 2, 3+ poor outcomes). Exposure to childhood maltreatment was significantly associated with a higher count of psychosocial and vocational disadvantage regardless of whether this was reported prospectively or retrospectively (Table S6). For the count of vocational disadvantage, this association was similar for prospective and retrospective reports. However, for the count of psychosocial disadvantage there was a significantly stronger association with retrospective compared to prospective reports, with retrospective reports of multiple (2 or more) forms of maltreatment yielding a particularly strong association.

Table S7 shows the associations between any childhood maltreatment and counts of age-18 psychosocial and vocational disadvantage taking into account whether or not there is a concordant maltreatment report type. Irrespective of whether maltreatment was reported only prospectively, only retrospectively, or both, this experience was associated with significantly higher counts of functional problems. For the count of vocational disadvantage, effect sizes were similar regardless of whether there was a corresponding report type or not. However, the count of psychosocial disadvantage was significantly higher for individuals with only retrospective reports compared to those with only prospective reports. Moreover, this count was lower for individuals with only a prospective report of maltreatment compared to those with both prospective and retrospective reports.

**Table S6**

*Associations of Prospective Informant-Reports and Retrospective Self-Reports of Childhood Maltreatment with Count of Age-18 Functional Outcomes.*

| **Maltreatment type** | **Report type ^a^** | **Association with age-18 poor functioning** | |
| --- | --- | --- | --- |
|  |  | **Psychosocial disadvantage** | **Vocational disadvantage** |
|  |  | b (95% CI) | b (95% CI) |
| Any maltreatment | Prospective | 0.90^***^ (0.50, 1.30) | 0.92^***^ (0.52, 1.33) |
|  | Retrospective | 2.43^***^ (2.09, 2.77) | 0.83^***^ (0.46, 1.20) |
| Multiple maltreatment ^b^ | Prospective |  |  |
|  | 0 | [reference] | [reference] |
|  | 1 | 0.69^**^ (0.25, 1.14) | 0.79^**^ (0.33, 1.25) |
|  | 2+ | 1.32^***^ (0.60, 2.04) | 1.18^**^ (0.45, 1.92) |
|  | Retrospective |  |  |
|  | 0 | [reference] | [reference] |
|  | 1 | 2.15^***^ (1.78, 2.52) | 0.73^**^ (0.32, 1.15) |
|  | 2+ | 3.32^***^ (2.65, 3.99) | 1.08^**^ (0.37, 1.79) |

*Note.* b=unstandardized coefficient, CI=confidence interval. ^*^*p*<.05, ^**^*p*<.01, ^***^*p*<.001. N=2,042. All analyses are adjusted for sex, family socioeconomic status, child IQ, and the non-independence of twin observations. ^a^ For report type, clear cells highlight the associations arising from prospective informant-reports of childhood maltreatment; grey shaded cells highlight the associations arising from retrospective self-reports of childhood maltreatment. ^b^ Multiple forms of maltreatment indicated two or more forms of maltreatment which could include physical abuse, sexual abuse, physical neglect and/or emotional abuse/neglect. Regression analyses used the ordinal multiple maltreatment variable, which ranged from 0 (no maltreatment) to 1 (one form of maltreatment) to 2+ (two or more forms of maltreatment).

**Table S7**

*Associations of the Concordance Between Prospective and Retrospective Reports of Any Childhood Maltreatment with a Count of Age-18 Functional Outcomes.*

| **Any maltreatment report concordance ^a^** | **Association with age-18 poor functioning** | |
| --- | --- | --- |
|  | **Psychosocial disadvantage** | **Vocational**  **disadvantage** |
|  | b (95% CI) | b (95% CI) |
| No maltreatment | [reference] | [reference] |
| Prospective report only | 0.68^**^ (0.27, 1.09) | 0.96^***^ (0.49, 1.43) |
| Retrospective report only | 2.43^***^ (2.07, 2.78) | 0.85^***^ (0.40, 1.30) |
| Both reports | 2.73^***^ (1.87, 3.58) | 1.13^**^ (0.49, 1.76) |
| No maltreatment | -2.73^***^ (-3.58, -1.87) | -1.13^**^ (-1.76, -0.49) |
| Prospective report only | -2.05^***^ (-2.95, -1.15) | -0.16 (-0.88, 0.55) |
| Retrospective report only | -0.30 (-1.19, 0.59) | -0.28 (-1.05, 0.49) |
| Both reports | [reference] | [reference] |

*Note*. b=unstandardized coefficient, CI=confidence interval. ^*^*p*<.05, ^**^*p*<.01, ^***^*p*<.001. N=2,042. All analyses are adjusted for sex, family socioeconomic status, child IQ, and the non-independence of twin observations. ^a^ Report concordance indicates the presence of maltreatment according to: neither prospective or retrospective reports (coded ‘0’), prospective report only (1), retrospective report only (2), both prospective and retrospective reports (3). Clear cells show coefficients with ‘no maltreatment’ as the reference category; grey shaded cells show coefficients with ‘both reports’ as the reference category.

***Are findings consistent when associations between retrospectively reported maltreatment and functional outcomes are adjusted for age-18 depressive disorder?***

We repeated our analyses of the association between retrospective self-reports of childhood maltreatment and functional outcomes and adjusted for age-18 depressive disorder (in addition to sex, child IQ, family socioeconomic status, and the non-independence of twin observations). Results are presented in Table S8. Elevated odds for psychosocial and vocational disadvantage were evident for individuals exposed to maltreatment regardless of report type. Adjusting for current depressive disorder lowered the odds (though not significantly) of psychosocial disadvantage for those who retrospectively reported maltreatment. Nonetheless, associations with psychosocial disadvantage typically remained significantly stronger for retrospective reports compared to prospective reports. The associations between childhood maltreatment and vocational disadvantage remained comparable for prospective and retrospective reports.

Table S9 shows the associations between childhood maltreatment and age-18 functional outcomes when the corresponding report type was also considered, adjusted for age-18 depressive disorder (in addition to sex, child IQ, family socioeconomic status, and the non-independence of twin observations). Once again, maltreatment remains associated with a higher likelihood of poor functioning regardless of whether this maltreatment was reported only prospectively, only retrospectively, or both. The magnitude of these associations was similar for vocational disadvantage but differed for psychosocial disadvantage. Individuals whose maltreatment was reported only retrospectively had significantly higher odds of poor psychosocial outcomes compared to those who had only prospective reports of maltreatment. There was also a trend towards those individuals with only prospective reports to have lower odds of psychosocial disadvantage relative to those whose who also had a concordant retrospective report.

**Table S8**

*Associations of Prospective Informant-Reports and Retrospective Self-Reports of Childhood Maltreatment with Age-18 Functional Outcomes ^a^*

| **Maltreatment type** | **Report type ^b^** | **Association with age-18 poor functioning** | |
| --- | --- | --- | --- |
|  |  | **Psychosocial disadvantage** | **Vocational disadvantage** |
|  |  | OR (95% CI) | OR (95% CI) |
| Any maltreatment | Prospective | 2.03^**^ (1.36, 3.04) | 2.19^***^ (1.42, 3.38) |
|  | Retrospective | 6.43^***^ (3.80, 10.87) | 1.75^**^ (1.20, 2.57) |
| Multiple maltreatment ^c^ | Prospective |  |  |
|  | 0 | [reference] | [reference] |
|  | 1 | 1.95^**^ (1.22, 3.12) | 1.88^**^ (1.17, 3.03) |
|  | 2+ | 2.20^*^ (1.05, 4.60) | 2.99^*^ (1.28, 6.98) |
|  | Retrospective |  |  |
|  | 0 | [reference] | [reference] |
|  | 1 | 5.60^***^ (3.26, 9.62) | 1.71^*^ (1.11, 2.63) |
|  | 2+ | 13.64^***^ (3.26, 57.14) | 1.89^†^ (0.91, 3.93) |

*Note.* CI=confidence interval, OR=odds ratio. ^†^*p*<.10, ^*^*p*<.05, ^**^*p*<.01, ^***^*p*<.001. N=2,032 for models predicting psychosocial disadvantage; N=2,019 for models predicting vocational disadvantage. ^a^ All analyses are adjusted for sex, family socioeconomic status, child IQ, and the non-independence of twin observations. In addition, analyses of the associations between retrospective self-reports of childhood maltreatment and functional outcomes are also adjusted for age-18 depressive disorder. ^b^ For report type, clear cells highlight the associations arising from prospective informant-reports of childhood maltreatment; grey shaded cells highlight the associations arising from retrospective self-reports of childhood maltreatment. ^c^ Multiple forms of maltreatment indicated two or more forms of maltreatment which could include physical abuse, sexual abuse, physical neglect and/or emotional abuse/neglect. Regression analyses used the ordinal multiple maltreatment variable, which ranged from 0 (no maltreatment) to 1 (one form of maltreatment) to 2+ (two or more forms of maltreatment).

**Table S9**

*Associations of the Concordance and Discordance Between Prospective and Retrospective Reports of Any Childhood Maltreatment with Age-18 Functional Outcomes ^a^*

| **Any maltreatment report concordance ^b^** | **Association with age-18 poor functioning** | |
| --- | --- | --- |
|  | **Psychosocial disadvantage** | **Vocational disadvantage** |
|  | OR (95% CI) | OR (95% CI) |
| No maltreatment | [reference] | [reference] |
| Prospective report only | 1.61^*^ (1.06, 2.44) | 2.20^**^ (1.37, 3.53) |
| Retrospective report only | 7.79^***^ (4.26, 14.26) | 1.78^**^ (1.16, 2.73) |
| Both reports | 4.05^*^ (1.48, 11.12) | 2.24^*^ (1.02, 4.92) |
| No maltreatment | 0.25^**^ (0.09, 0.68) | 0.45^*^ (0.20, 0.98) |
| Prospective report only | 0.40^†^ (0.14, 1.15) | 0.98 (0.42, 2.30) |
| Retrospective report only | 1.92 (0.60, 6.16) | 0.80 (0.33, 1.91) |
| Both reports | [reference] | [reference] |

*Note*. CI=confidence interval, OR=odds ratio. ^a^ All analyses are adjusted for sex, family socioeconomic status, child IQ, age-18 depressive disorder, and the non-independence of twin observations. ^†^*p* <.10, ^*^*p*<.05, ^**^*p*<.01, ^***^*p*<.001. ^b^ Report concordance indicates the presence of maltreatment according to: neither prospective or retrospective reports (coded ‘0’), prospective report only (1), retrospective report only (2), both prospective and retrospective reports (3). Clear cells show ORs with ‘no maltreatment’ as the reference category; grey shaded cells show ORs with ‘both reports’ as the reference category.

**References**

1. Trouton A, Spinath FM, Plomin R (2002) Twins early development study (TEDS): a multivariate, longitudinal genetic investigation of language, cognition and behavior problems in childhood. Twin Res 5:444-448. https://doi.org/10.1375/twin.5.5.444
2. Moffitt TE, E-Risk Study Team (2002) Teen-aged mothers in contemporary Britain. J Child Psychol Psychiatry 43:727–742. https://doi.org/10.1111/1469-7610.00082
3. CACI Information Services, 2006. ACORN user guide. CACI, London, UK.
4. Caspi A, Taylor A, Moffitt TE, Plomin R (2000) Neighborhood deprivation affects children's mental health: Environmental risks identified in a genetic design. Psychol Sci 11:338-342. https://doi.org/10.1111%2F1467-9280.00267
5. Jaffee SR, Caspi A, Moffitt TE, Taylor A (2004) Physical maltreatment victim to antisocial child: evidence of an environmentally mediated process. J Abnorm Psychol 113:44-55. https://doi.org/10.1037/0021-843X.113.1.44
6. Jaffee SR, Caspi A, Moffitt TE, Polo-Tomas M, Taylor A (2007) Individual, family, and neighborhood factors distinguish resilient from non-resilient maltreated children: a cumulative stressors model. Child Abuse Negl 31:231–253. https://doi.org/10.1016/j.chiabu.2006.03.011

Danese A, Moffitt TE, Arseneault L, Bleiberg BA, Dinardo PB, Gandelman SB, Houts R, Ambler A, Fisher HL, Poulton R (2017) The origins of cognitive deficits in victimized children: implications for neuroscientists and clinicians. Am J Psychiatry 174:349-361. https://doi.org/10.1176/appi.ajp.2016.16030333

1. Fisher HL, Caspi A, Moffitt TE, Wertz J, Gray R, Newbury J, Ambler A, Zavos H, Danese A, Mill J, Odgers CL, Pariante C, Wong CCY, Arseneault L (2015) Measuring adolescents’ exposure to victimization: The Environmental Risk (E-Risk) longitudinal twin study. Dev Psychopathol 27:1399–1416. https://doi.org/10.1017/S0954579415000838

Bradley R, Caldwell B (1977) Home observation for measurement of the environment: a validation study of screening efficiency. Am J Ment Defic 81:417-420.

1. Dodge KA, Bates JE, Pettit GS (1990) Mechanisms in the cycle of violence. Science 250:1678-1683. https://doi.org/10.1126/science.2270481
2. Lansford JE, Dodge KA, Pettit GS, Bates J E, Crozier J, Kaplow J (2002) A 12-year prospective study of the long-term effects of early child physical maltreatment on psychological, behavioral, and academic problems in adolescence. Arch Pediatr Adolesc Med 156:824-830. https://doi.org/doi:10.1001/archpedi.156.8.824
3. Goldman‐Mellor S, Caspi A, Arseneault L, Ajala N, Ambler A, Danese A, Fisher HL, Hucker A, Odgers C, Williams T, Wong C, Moffitt TE (2016) Committed to work but vulnerable: self‐perceptions and mental health in NEET 18‐year olds from a contemporary British cohort. J Child Psychol Psychiatry 57:196-203. https://doi.org/10.1111/jcpp.12459
4. Office for National Statistics (2013) UK Estimate of Young People Not in Education, Employment or Training. London, UK.
5. Office for National Statistics (2014) Young People Not in Education, Employment or Training (NEET). London, UK.

Office for National Statistics (2016) Childbearing for women born in different years. London, UK.

1. Finkelhor D, Hamby SL, Turner HA, Ormrod RK (2011) The Juvenile Victimization Questionnaire: 2nd Revision (JVQ-R2) Durham, NH: Crimes Against Children Research Center.
2. Hamby S, Finkelhor D, Ormrod D, Turner H (2004) The comprehensive JVQ administration and scoring manual. Durham, NH: University of New Hampshire, Crimes Against Children Research Centre.
3. Caspi A, Moffitt TE, Thornton A, Freedman D, Amell JW, Harrington H, Smeijers J, Silva PA (1996) The life history calendar: a research and clinical assessment method for collecting retrospective event-history data. Int J Meth Psychiatr Res 6:101-114.

Zimet GD, Dahlem NW, Zimet SG, Farley GK (1988) The Multidimensional Scale of Perceived Social Support. J Pers Assess 52:30-41. https://doi.org/10.1207/s15327752jpa5201_2

Diener E, Emmons RA, Larsen RJ, Griffin S (1985) The Satisfaction with Life Scale. J Pers Assess 49:71-75. https://doi.org/10.1207/s15327752jpa4901_13

Russell DW (1996) UCLA Loneliness Scale (Version 3): reliability, validity, and factor structure. J Pers Assess 66:20–40. https://doi.org/10.1207/s15327752jpa6601_2

1. Hughes ME, Waite LJ, Hawkley LC, Cacioppo JT (2004) A short scale for measuring loneliness in large surveys: results from two population-based studies. Res Aging 26:655–672. https://doi.org/10.1177%2F0164027504268574

Buysse DJ, Reynolds CF, Monk TH, Berman SR, Kupfer DJ (1989) The Pittsburgh Sleep Quality Index: a new instrument for psychiatric practice and research. Psychiatry Res 28:193–213.
